# Synthesis of Platinum Nanoparticles Supported on Fused Nanosized Carbon Spheres Derived from Sustainable Source for Application in a Hydrogen Generation Reaction

**DOI:** 10.3390/nano13131994

**Published:** 2023-07-01

**Authors:** Erik Biehler, Qui Quach, Tarek M. Abdel-Fattah

**Affiliations:** 1 Applied Research Center, Thomas Jefferson National Accelerator Facility, Newport News, VA 23606, USA; erik.biehler.16@cnu.edu (E.B.); qui.quach.13@cnu.edu (Q.Q.); 2 Department of Molecular Biology and Chemistry, Christopher Newport University, Newport News, VA 23606, USA

**Keywords:** hydrogen, nanoparticles, carbon, catalysis, sustainability, energy, green chemistry, environment, metals, platinum

## Abstract

The dwindling supply of fossil fuels has prompted the search for an alternative energy source that could effectively replace them. Potential renewable energy sources such as solar, wind, tidal, and geothermal are all promising but each has its own drawbacks. Hydrogen gas on the other hand can be combusted to produce energy with only water as a byproduct and can be steadily generated via the aqueous media hydrolysis reaction of Sodium Borohydride (NaBH_4_). This study successfully synthesized fused carbon spheres derived from sugar and decorated them with platinum nanoparticles to form a novel composite material (PtFCS) for catalyzing this reaction. The platinum nanoparticles were produced by reducing chloroplatinic acid in a solution with sodium borohydride and using sodium citrate as a capping agent for the nanoparticles. Transmission electron microscopy (TEM), Energy-dispersive X-ray spectroscopy (EDS), Fourier-transform infrared spectroscopy (FTIR), and X-ray diffraction (XRD) were used to characterize and determine the size and shape of the Pt nanoparticles (PtNPs) and fused carbon spheres. TEM was able to determine the average size of the fused carbon spheres to be 200 nm and the average size for the PtNPs to be 2–3 nm. The PtFCS composite was tested for its ability to catalyze the hydrolysis of NaBH_4_ under various reaction conditions including various solution pH, various temperatures, and various dosages of sodium borohydride. The catalyst was found to perform the best under acidic solution conditions (pH 6), producing hydrogen at a rate of 0.0438 mL/mg_cat_·min. The catalyst was determined to have an activation energy of 53.0 kJ/mol and could be used multiple times in succession with no loss in the volume of hydrogen produced. This sugar-derived composite catalyst shows promise and could be implemented as a sustainable catalyst for the generation of hydrogen fuel.

## 1. Introduction

It is no secret that the world is overly reliant on fossil fuels as an energy source with an estimated 84.3% of all global energy being derived from these limited and environmentally damaging fuels [1]. The combustion of these fuels is the number one source of greenhouse gases such as carbon dioxide (CO_2_), and some models predict that the reserves of coal, oil, and natural gas may be depleted in as little as 105, 35, and 37 years, respectively [2,3]. Thus, there has been a considerable global research effort that has focused on developing an alternative energy source that is not only clean but also abundant. There are multiple promising candidates for renewable energy including solar, wind, tidal, geothermal, and hydrogen, each with its own set of benefits and drawbacks [4,5,6,7,8,9,10,11,12,13,14]. Solar energy, for example, is a promising candidate as energy from the sun is the most abundant energy source we have currently. Direct solar energy can be used to boil water, cook food, or even dry food for storage, replacing the fossil fuels that are more typically used [5]. Solar panels have gained traction as a way to convert photovoltaic energy into electricity; however, current solar panels are often expensive to produce and lack efficiency [6]. Wind energy is another renewable source that is abundant and, through modern technology, has managed to become relatively free of pollutants [7]. Opponents of wind energy point out that the development of wind farms has led to an increase in noise pollution and that wind turbines have disrupted the flight patterns of some bird species resulting in deaths from collisions [8]. Tidal energy is a relatively new renewable energy source that is promising due to it being one of the most predictable forms of renewable energy [9]. The main concern with tidal energy is that there have not yet been enough studies on the environmental impacts. There are concerns over the impacts the construction of tidal energy devices will have on local species, as well as economic concerns over how these structures will affect shipping routes [9]. Geothermal energy is another energy source that is highly consistent like tidal energy; however, the main drawbacks of geothermal energy are inefficiency compared to other renewable energy sources and a high initial cost to implement [10,11]. Hydrogen, which has an atomic number of 1, is the most abundant element in the universe and primarily exists in a diatomic gaseous state. Hydrogen gas is unique not only in that it can be combusted to produce energy, but that there are no harmful byproducts produced via this reaction, only water. This abundance and environmentally friendly bioproduct make hydrogen gas an extremely promising choice as a renewable energy source to replace the world’s reliance on fossil fuels. Hydrogen gas, however, is not without its drawbacks as the main issue standing in the way of a widely implemented hydrogen fuel economy lies in the struggle to efficiently store it. There are two main methods of hydrogen storage, first, keeping hydrogen in its diatomic gaseous state and compressing the gas into tanks, and second, cooling the gas down into a liquid to be stored in refrigerated tanks. The first method is relatively simple, however, there are safety concerns associated with compressing the highly combustible gas. The refrigeration technique also is not ideal as the energy cost to cool and store the gas would essentially offset the benefits of using the fuel. A different way to store hydrogen that has gained attention in recent years is storage within the structure of a chemical species. A class of chemicals known as metal hydrides is capable of storing large percentages of hydrogen in their structure. Sodium borohydride (NaBH_4_), for example, contains 10.8% hydrogen by weight and when mixed in and reacted with water, produces hydrogen gas steadily over time (1) [15]. The main drawback of this reaction, however, is that the gas is produced too slowly to be effectively utilized.

(1) $$NaBH_{4}+2H_{2}O\to NaBO_{2}+4H_{2}$$

Transition metals have been extensively studied for their catalytic ability that stems from their incomplete valence shells and their ability to transfer electrons. Precious metal catalysts in particular have been studied for their catalytic ability and this team as well as others have explored catalysts made from these metals including gold, silver, palladium, and platinum to make this reaction more efficient [16,17,18,19,20,21,22,23]. Nanomaterials made using these metals and more common metals such as zinc have been used for hydrogen generation [24,25,26,27,28,29], antibacterial effects [30], gas detection [31], optoelectronic properties [32], and as catalyst [33,34]. Platinum metal, in particular, is commonly used as a catalyst in its bulk state for hydrosilation reactions [22], oxidation of methane [35], and in catalytic converters [36], while as nanoparticles (PtNPs), platinum has been used as a catalyst for the hydrogenation of alkynes [37], in hydrogen fuel cells [38], and in hydrogen generation reactions [18,39]. Metal nanoparticles make excellent catalysts due to their increased surface area over bulk materials; however, it has been noted that nanoparticles have a tendency to agglomerate in solutions, decreasing their catalytic effectiveness [40]. A way to mitigate this agglomeration is through the use of a support material, which gives the nanoparticles a surface to disperse on and bind to rather than each other. There are different support materials available, however, support materials made from carbon are an attractive option due to their high surface area and tensile strength [41]. Additionally, there is an environmentally friendly aspect to using carbon over other materials, since carbon is regularly found in the environment, and structures made of it have an easier time breaking down. In this study, we aimed to synthesize and characterize a novel catalyst comprised of platinum nanoparticles supported on a fused carbon sphere composite (PtFCS). This material was then tested for its effectiveness as a catalyst in the water-splitting reaction of sodium borohydride under a variety of different reaction conditions including different pH conditions, solution temperature conditions, and doses of NaBH_4_ added to the solution. The activation energy of this reaction as catalyzed by PtFCS was then able to be determined and was compared to other catalysts used in the hydrolysis of sodium borohydride.

## 2. Experimental

### 2.1. Synthesis of Platinum Nanoparticles

The platinum nanoparticles (PtNPs) were synthesized via the reduction of chloroplatinic acid (Cl_6_H_2_Pt). Then, 48 mL of 10 mM beta cyclodextrin solution was mixed with 1 mM of chloroplatinic acid. The mixture was stirred for 30 min. After that, 0.25 mL of 180 mM sodium borohydride (NaBH_4_) was added slowly to the mixture. The solution was stirred for 2 h. The nanoparticle solution was centrifuged at 10,000 rpm for 15 min to remove unreacted reactants.

### 2.2. Synthesis of Fused Carbon Spheres and Nanocomposite Catalyst

In order to synthesize the fused carbon sphere support material, dextrose was first dissolved into deionized water resulting in a solution with a 0.5 M concentration. The dextrose solution was then poured into the polytetrafluoroethylene inside the body of a stainless-steel reaction vessel so that there was a 3:2 ratio of air to dextrose solution. The stainless-steel reaction vessel containing the solution was then placed into an oven which was heated to 473 K and left overnight, which resulted in the desired fused carbon spheres (FCS). Once the fused carbon spheres had been formed, the solution was filtered via vacuum filtration to collect all solid material. The solid material was then washed several times with deionized water and left out at room temperature to dry.

The fused carbon sphere composites were produced by incipient wetness impregnation of 100 mg of fused carbon spheres by 2 mL of the PtNPs aqueous solution. First, the dried fused carbon spheres were placed into a small beaker. Next, the nanoparticle solution was poured over the top of the powdered fused carbon spheres and stirred well at room temperature. The resulting mixture was then stored at 333 K for two days to facilitate the evaporation of excess water from the composite material. After the two days had passed, the dried material was collected and stored until needed for characterization or the catalytic trials.

### 2.3. Characterization

The PtFCS material was first characterized via transmission electron microscopy (TEM, JEM-2100F) to confirm the adhesion of the nanoparticles to the surface of the spheres as well as to confirm the final morphology of the nanoparticle-coated fused carbon sphere composite.

Energy-dispersive X-ray spectroscopy (EDS, ThermoScientific UltraDry, Waltham, MA, USA) allowed us to determine what elements were present within the chemical structure of the catalyst. The surface structure of the material after reusability trials was shown by scanning electron microscopy (SEM, ThermoScientific UltraDry, Waltham, MA, USA).

Fourier-transform infrared spectroscopy (FTIR, Shimadzu IR-Tracer 100, Kyoto, Japan) allowed us to determine any functional groups present in the catalyst and supported the identification of the nanoparticles. Finally, powder X-ray diffraction (P-XRD, Rigaku MiniFlex II Benchtop X-ray Diffractometer, Tokyo, Japan) was implemented in order to further confirm any chemical species within our material.

### 2.4. Catalysis

The setup consisted of two vacuum flasks connected to one another by a thin plastic tube. One flask was designated as the reaction chamber inside of which the hydrolysis reaction of sodium borohydride catalyzed by platinum-decorated fused carbon sphere composites occurred. The second flask contained only deionized water, which would be displaced by the hydrogen generated in the first flask. Both flasks were sealed via rubber stoppers, however, the second flask, containing the DI water to be displaced, included a second thin plastic tube that ran through the rubber stopper that sealed the second flask. This second hose then hung over the mouth of a plastic cup that was placed on a microbalance scale. The scale was then balanced and any water that was displaced by hydrogen gas would drip into the cup so that the mass could be measured. When sodium borohydride was added to the first flask and carefully sealed, hydrogen gas would begin to fill the first flask. Once that flask was full of hydrogen gas, the gas would move through the thin plastic tubing that connected the two flasks. As the gas filled the second flask, the deionized water present in the flask would be forced up the thin plastic tube that was going through the rubber stopper and would begin filling the cup on the micro balance scale. This scale was connected to a laboratory computer and a measurement program was run, which recorded the measured mass of the water displaced every 0.25 s. This experiment was run at a variety of pH levels (6, 7, 8), temperatures (283, 288, 295, 303) K, and NaBH_4_ doses (625, 925, 1225) μmoles to determine optimal reaction conditions. All reactions were stirred using a magnetic stir bar for the full two hours of the trial except in the cases of the temperature-controlled trials, which required insulation that interfered with the magnetic stir plate.

## 3. Results and Discussion

**Figure 1:**
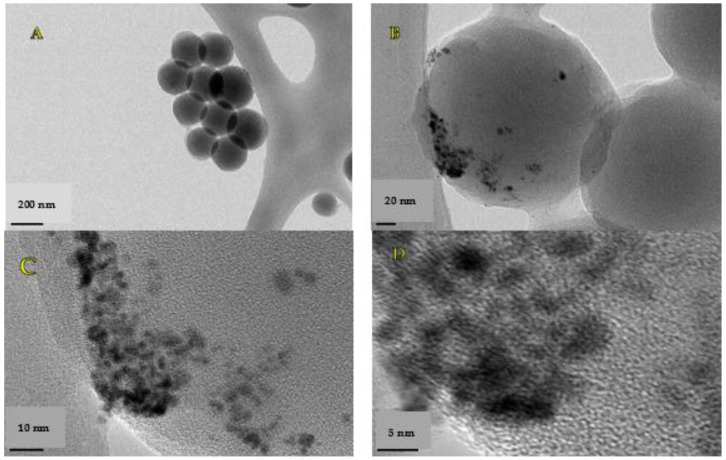
TEM Images of the PtFCS composite material at a scale of 200 nm (**A**), 20 nm (**B**), 10 nm (**C**), 5 nm (**D**).

The novel PtFCS catalyst was characterized via transition electron microscopy as shown in Figure 1. Figure 1A depicts the structure of the fused carbon sphere backbone as a collection of semi-round fused objects with a diameter ranging from about 180 to 250 nm. Zooming in on these spheres revealed the presence of nanoparticles, which can be seen in Figure 1B–D. The nanoparticles can be seen spread across the fused carbon spheres in Figure 1B or in more concentrated groups as in Figure 1C,D. From these images, the average size of the nanoparticles was determined to be about 2.9 nm. Despite some grouping, it is clear from these images that the platinum nanoparticles are dispersed across the materials, showing the fused carbon sphere successfully prevented major agglomeration.

**Figure 2:**
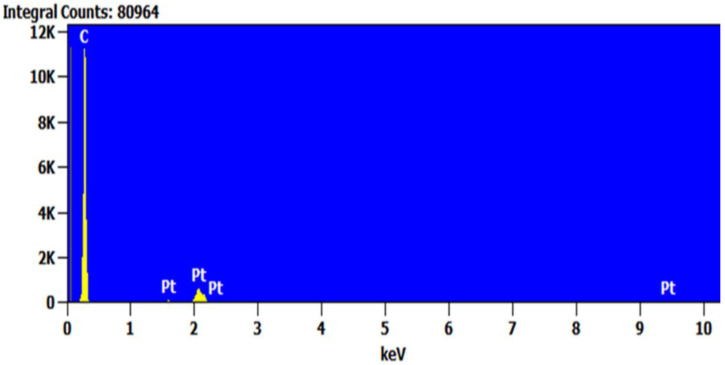
EDS spectra for PtFCS material.

After TEM, the PtFCS catalyst was characterized using EDS (Figure 2). The two main elements of the PtFCS were carbon and platinum. Carbon was the most abundant element in the composite material as the fused carbon sphere backbone is derived from carbon-based dextrose. the platinum peaks had a low number of counts; however, this is most likely due to the small nanoparticle size and the dispersion of nanoparticles across the fused carbon sphere backbone. The percentage of Pt loading is 1.57% ± 0.5 (Wt%).

**Figure 3:**
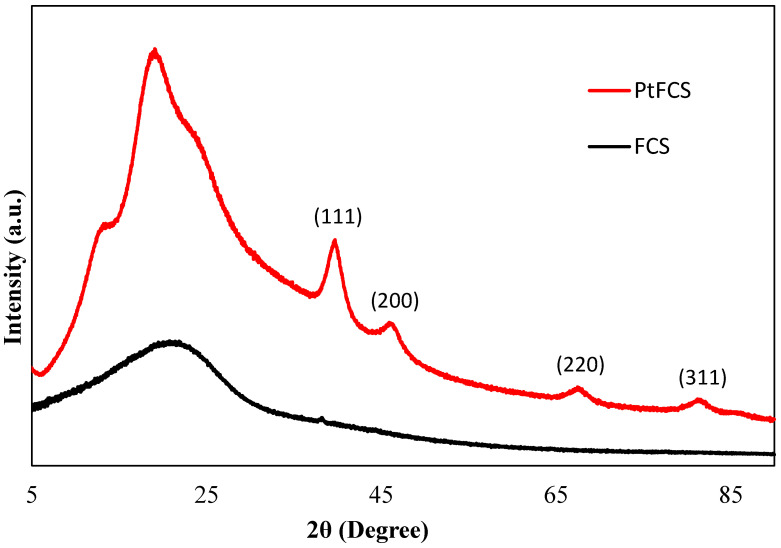
P-XRD analysis of PtFCS and FCS fused carbon sphere material.

PtFCS as well as fused carbon spheres with no nanoparticles on them were characterized via XRD analysis (Figure 3). A broad peak seen around 23 degrees was observed in the fused carbon sphere material, which is indicative of the graphitic characteristics of carbon-based materials [42]. Expectedly, this peak was also seen in the PtGLM composite since the fused carbon sphere support makes up a majority of the catalyst. The remaining peaks seen around 39.9°, 46.4°, 67.9°, and 81.6° all correspond to the (111), (200), (220), and (311) planes for the face-centered cubic structure of platinum nanoparticles (ICDD PDF 70-2431). The EDS spectra from Figure 2 confirm that there is platinum within the sample, and the TEM images from Figure 1 showed small nanoparticles; therefore, this XRD analysis further confirms that the metal present on the support material is platinum nanoparticles.

**Figure 4:**
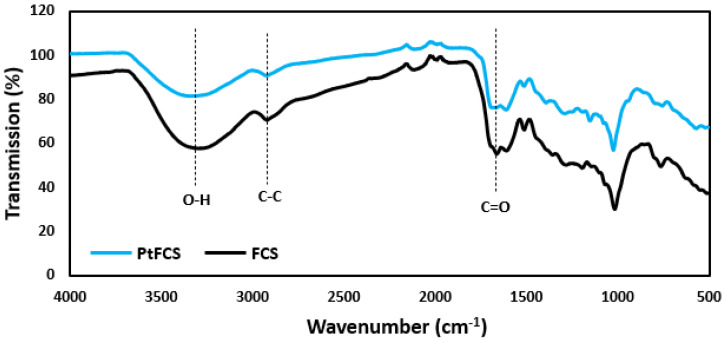
FTIR analysis of PtFCS and FCS fused carbon sphere material.

The final method of characterization of the PtFCS material was through FTIR analysis (Figure 4). Fused carbon nanospheres that contained no nanoparticles were also analyzed for comparison purposes. There did not appear to be not much of a difference in the locations of the peaks seen in the two materials, which could indicate that the addition of nanoparticles did not significantly change the fused carbon sphere structure. Three major peaks were seen in the materials that are indicative of the dextrose used to prepare the fused carbon spheres. The broad stretch from 3600–3000 cm^−1^, the small peak at 2900 cm^−1^, and the peak at 1700 cm^−1^ represent the hydroxyl (OH), alkane (C-C), and carbonyl (C=O) functional groups of dextrose.

**Figure 5:**
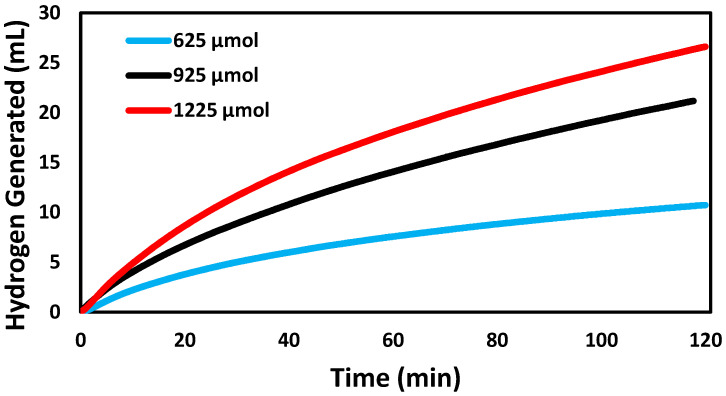
PtFCS catalyzed hydrolysis of NaBH_4_ at various doses.

The novel PtFCS composite was tested for its catalytic ability at NaBH_4_ doses of 625 μmol, 925 μmol, and 1225 μmol (Figure 5). At a dose of 625 μmol, the volume of hydrogen produced by the reaction was observed to be 10.7 mL, resulting in a generation rate of 0.0089 mL/mg_cat_·min for the two-hour trial. The dose of the NaBH_4_ was then raised to 925 μmol which increased the volume of hydrogen generated and the generation rate to 21.2 mL and 0.0180 mL/mg_cat_·min, respectively. Finally, the dose was further raised to 1225 μmol the volume and rate further increased to 26.6 mL and 0.0222 mL/mg_cat_·min. It is clear from these data that as the dosage of NabH_4_ is increased, so did the amount of hydrogen generated from the reaction. Based on Equation (1), this result follows the equilibrium law as the increase in the dose of a reactant shifts the reaction to produce more product.

**Figure 6:**
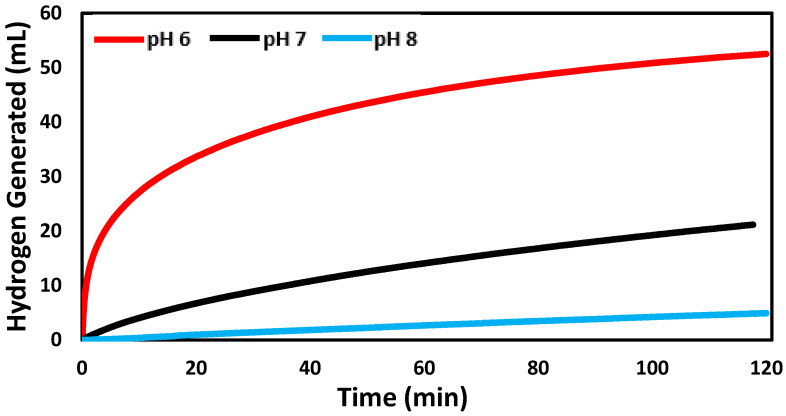
PtFCS catalyzed hydrolysis of NaBH_4_ at various pH’s.

PtFCS was next tested for its catalytic ability under acidic conditions (pH 6), neutral conditions (pH 7), and basic conditions (pH 8) (Figure 6). The reaction under acidic conditions or pH 6 was observed to produce 52.5 mL of hydrogen gas at a rate of 0.0438 mL/mg_cat_·min. A solution pH of 7 or neutral conditions resulted in a hydrogen generation rate of 0.0180 mL/mg_cat_·min and a volume of 21.2 mL of hydrogen gas. When the pH of the solution was raised to a basic pH of 8, a decrease in hydrogen generation was observed, with only 4.9 mL of hydrogen gas being generated after two hours of reaction time, resulting in a reaction rate of 0.0041 mL/mg_cat_·min. Based on these results, it is evident that the higher the pH of the reaction solution, the less hydrogen gas would be generated and that the opposite would occur for lower pHs. These results are consistent with the work of previous teams that have explored this reaction [15,35].

**Figure 7:**
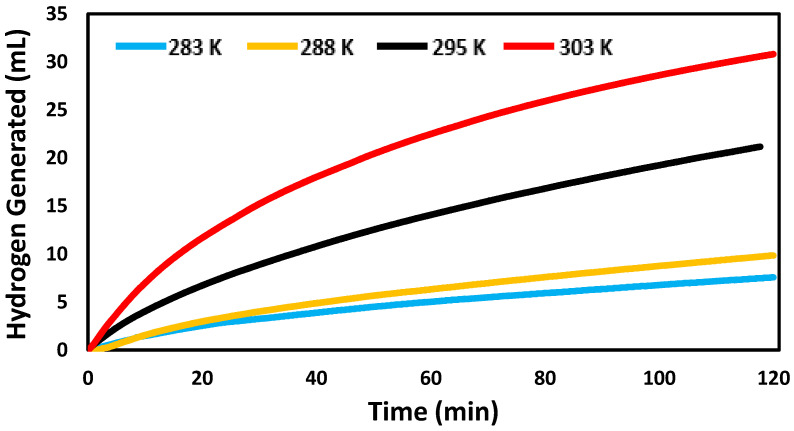
PtFCS catalyzed hydrolysis of NaBH_4_ at varying temperatures.

The catalytic ability of PtFCS to hydrolyze NaBH_4_ was also tested at the following temperatures: 283 K, 288 K, 295 K, and 303 K (Figure 7). The temperature of the reaction was first raised to 303 K, which resulted in a volume of hydrogen produced to be 30.8 mL at a rate of 0.0257 mL/mg_cat_·min. At room temperature (295 K), the reaction produced 21.2 mL of hydrogen gas at a rate of 0.0180 mL/mg_cat_·min. When cooled to 288 K, the hydrogen generation rate slowed to 0.0082 mL/mg_cat_·min producing only 9.8 mL of hydrogen gas. Finally, when the reaction solution was further cooled to 283 K, the reaction appeared to slow even further, producing only 7.6 mL of hydrogen gas at a rate of 0.0063 mL/mg_cat_·min. These results show a direct relationship between the temperature of the reaction solution and the volume of hydrogen gas that was produced. Since higher temperatures resulted in more hydrogen gas being produced, it was determined that this reaction is endothermic.

Once the temperature study (Figure 7) had been completed, the data could be used to find the activation energy of the reaction as catalyzed by PtFCS. Each temperature tested was entered into the Arrhenius Equation (2) where k is equal to the rate constant of the reaction at each temperature. A is equal to the pre-exponential factor, Ea is the activation energy of the reaction, R is the universal gas constant, and lastly, T represents the temperature tested.

(2) $$k=Ae^{-\frac{Ea}{RT}}$$

**Figure 8:**
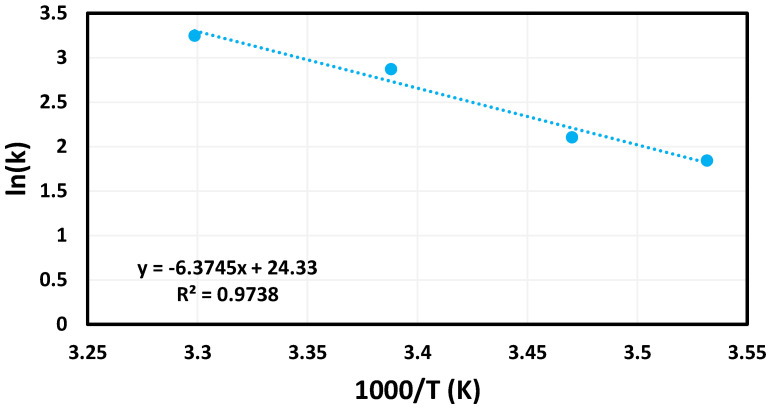
The Arrhenius plot created from the temperature data and the Arrhenius Equation (2).

The natural log of the rate constant (k) of the reaction at each tested temperature (T) was then plotted against 1000 divided by that temperature to create an Arrhenius plot (Figure 8). The equation of the line from this plot allowed for the activation energy of this reaction as catalyzed by PtFCS to be calculated as 53.0 kJ/mol. This activation energy was then compared to other catalysts for this reaction as seen in Table 1.

When compared to other catalysts used for this hydrolysis reaction (Table 1), PtFCS shows relatively comparable activation energy. It has lower activation energy than bulk metal catalysts such as nickel, as well as certain transition metal composite catalysts such as Ag/MWCNTs, however, it is not as low as others. Despite this relatively average activation energy, many of the catalysts that have a lower activation energy use support materials that are not as sustainable as fused carbon spheres, since fused carbon spheres can be derived simply from dextrose.

**Figure 9:**
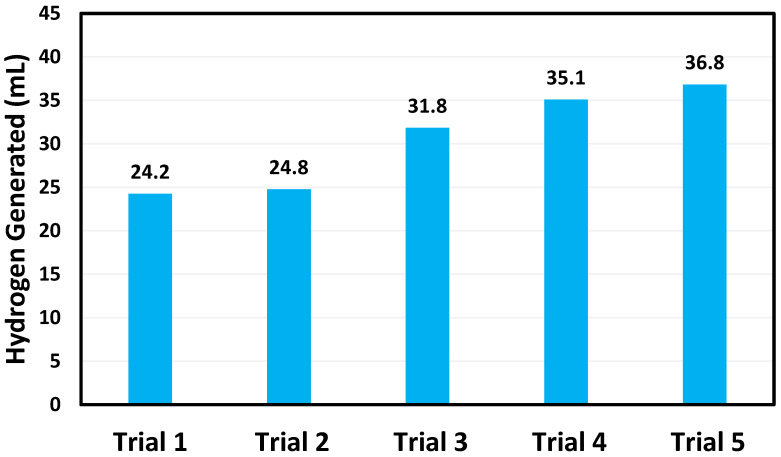
Catalytic reusability of PtFCS after five consecutive trials.

The final catalytic study performed on PtFCS was its ability to be used multiple times consecutively. A standard trial was begun at pH 7, 295 K, and using 925 μmol of NaBH_4_ which was run for a full two hours with magnetic stirring. After the first two hours had been completed, an additional 925 μmol NaBH_4_ was added to the reaction vessel and quickly closed, marking the start of a second two-hour trial. This same method was then repeated for an additional three trials for a total of five trials (Figure 9). Across the five trials, there was an observed average volume of 30.5 mL of hydrogen gas produced per trial. It was noted that the first two trials produced similar volumes of hydrogen gas with each subsequent trial increasing amounts. One possible explanation is that the platinum nanoparticle supported on the fused carbon sphere catalyst was becoming more catalytically activated with each subsequent trial, a phenomenon previously reported by Deraedt et. al. 2014 [54]. They theorized that the BH_4_^−^ forms strong hydridic bonds with the nanoparticles, stabilizing them and allowing for further catalyzation over the long term. After the reusability trials, the catalyst was collected and examined by P-XRD, FTIR, and SEM-EDS.

The P-XRD spectra of PtFCS after reusability experiments are shown in Supplementary Materials Figure S1. The broad peaks at 23° indicated the graphitic characteristics of carbon-based materials. The peaks at 39°, 46°, 67°, and 81°, which correspond to the (111), (200), (220), and (311) lattice planes of platinum nanoparticles were smaller. As discussed above, it could be due to BH_4_^−^ forming strong bonds with nanoparticles, which affected the crystallinity structure. The inset of Figure S1 indicated the P-XRD of sodium borohydride. The signature peaks of sodium borohydride at 29°, 41°, and 48° were observed on the PtFCS after reusability trials [55].

The Supplementary Materials Figures S2 and S3 indicate the FTIR of sodium borohydride and PtFCS after reusability trials. Both B-H bending and stretching functional groups at 1327 cm^−1^, 1095 cm^−1^, and 2276 cm^−1^ were observed on the FTIR spectra of PtFCS. The functional groups O-H, C-C, and C=O of PtFCS were still retained after reusability trials.

The surface structure of PtFCS after reusability trials is shown in Figure S4. Through the mapping elements on the surface, it was observed that the distribution of boron was matched with that of platinum. It highly supported BH_4_^−^ surrounding and stabilizing the platinum nanoparticles, which maintained their catalytic efficiency.

**Scheme 1:**
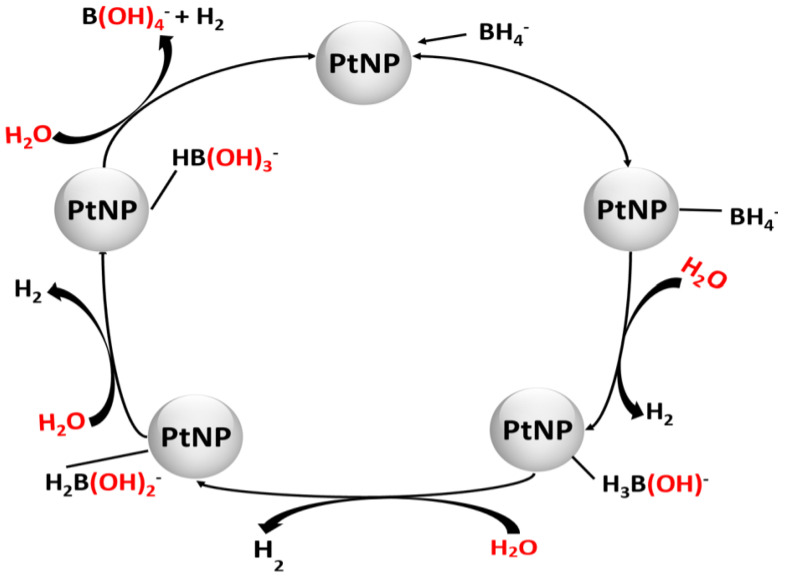
Hydrolysis of NaBH_4_ by PtFCS.

A proposed mechanism for the hydrolysis of NaBH_4_ by the PtFCS catalyst is depicted in Scheme 1. First, a borohydride ion (BH_4_^−^) attaches itself to a platinum nanoparticle resting on the surface of the fused carbon sphere material. A nearby water molecule then attacks the boron from the borohydride and splits, leaving a hydroxyl group and releasing a hydrogen gas molecule. This can happen up to three more times, releasing four diatomic gas molecules in total. After the fourth hydrogen gas molecule is produced, the remaining tetrahydroxyborate [B(OH)_4_] molecule can detach from the platinum nanoparticle and allow for another BH_4_^−^ to take its place.

## 4. Conclusions

In conclusion, the successful synthesis of the novel PtFCS composite was confirmed through the use of FTIR, XRD, TEM, and EDS analysis. The catalyst was tested at various temperatures, pH conditions, and doses of NaBH_4_ revealing that the reaction has an activation energy of 53.0 kJ/mol, which is competitive compared to similar catalysts. This catalyst produced the most hydrogen under the reaction conditions of pH 6, a temperature of 295 K, and a dosage of 925 μmol of NaBH_4_. When tested for its structural stability, it was found that the same amount of catalyst could be used at least five times in a row with increasing volumes of hydrogen being produced with later trials. This could indicate that with each use, the catalyst becomes further activated. Since the fused carbon sphere backbone is synthesized from dextrose and the catalyst can be used multiple times without a decrease in hydrogen production, this novel catalyst shows promise as a sustainable way to produce hydrogen gas. This work can be expanded upon in a few ways. First, the conditions we already tested could be taken further, i.e., more temperatures and more pHs. Additionally, different metals such as gold, silver, and palladium could be tested to see how composite catalysts made from these metals compare to this metal and previous materials.

## Figures and Tables

**Table 1:** Comparison of Activation energies for the catalyzed hydrolysis of NaBH_4_.

Catalyst	E_a_ (kJ mol^−1^)	Temperature (K)	Reference
Ni	71	273–308	[43]
Raney-Nickel	63	273–308	[43]
Co	75	273–308	[43]
Co/MWCNTs	63.8	303–318	[44]
CoB-zeolite-HCl	42.5	293–323	[45]
MCCC	64.3	303	[46]
Pt–Pd/CNTs	19	302–332	[47]
Au/MWCNTs	21.1	273–303	[48]
Ag/MWCNTs	44.5	273–303	[17]
Pd/MWCNTs	62.7	273–303	[19]
BCD-AuNP	54.7	283–303	[16]
PtNPs	39.2	283–303	[18]
Pd Nanocup	58.9	283–303	[49]
AgNPs	50.3	273–303	[50]
AgNP-FCS	37.0	273–303	[51]
Pt/C	45	298–313	[52]
PGO	N/A	298	[53]
PtFCS	53.0	283–303	This Work

## Data Availability

The data presented in this study are available on request from the corresponding author.

## Supplementary Materials

The following supporting information can be downloaded at: https://www.mdpi.com/article/10.3390/nano13131994/s1, Figure S1: P-XRD of PtFCS after reusability trials. The inset showed the P-XRD of sodium borohydride. The red color showed the signature peaks of sodium borohydride. Figure S2: FTIR of sodium borohydride (NaBH4). Figure S3: FTIR of PtFCS after reusability trials. Figure S4: (a) SEM of PtFCS after reusability trials; (b) mapping of boron (B); (c) mapping of carbon (C); (d) mapping of platinum (Pt).

## References

[1] Ritchie H., Roser M. (2014). Energy-Our World in Data. https://ourworldindata.org/energy.

[2] Rodhe H.A. (1990). Comparison of the Contribution of Various Gases to the Greenhouse Effect. Science.

[3] Shafiee S., Topal E. (2009). When Will Fossil Fuel Reserves Be Diminished?. Energy Policy.

[4] Panwar N.L., Kaushik S.C., Kothari S. (2011). Role of renewable energy sources in environmental protection: A review. Renew. Sustain. Energy Rev..

[5] Tucker M. (1999). Can solar cooking save the forests?. Ecol. Econ..

[6] Adamson A.W., Namnath J., Shastry V.J., Slawson V. (1984). Thermodynamic inefficiency of conversion of solar energy to work. J. Chem. Educ..

[7] Ottinger R.L. Experience with promotion of renewable energy: Successes and lessons learned. Proceedings of the Parliamentarian Forum on Energy Legislation and Sustainable Development.

[8] US Government Accountability Office (GOA) (2005). Wind Power: Impacts on Wildlife and Government Responsibilities for Regulating Development and Protecting Wildlife.

[9] O’Doherty T., O’Doherty D.M., Mason-Jones A. (2018). Tidal Energy Technology. Wave Tidal Energy.

[10] Anderson A., Rezaie B. (2019). Geothermal technology: Trends and potential role in a sustainable future. Appl. Energy.

[11] Li K., Bian H., Liu C., Zhang D., Yang Y. (2015). Comparison of geothermal with solar and wind power generation systems. Renew. Sustain. Energy Rev..

[12] Daut I., Razliana A.R.N., Irwan Y.M., Farhana Z. (2012). A Study on the Wind as Renewable Energy in Perlis, Northern Malaysia. Energy Procedia.

[13] Kabir E., Kumar P., Kumar S., Adelodun A.A., Kim K.-K. (2018). Solar energy: Potential and future prospects. Renew. Sustain. Energy Rev..

[14] Bosnjakovic M., Sinaga N. (2020). The Perspective of Large-Scale Production of Algae Biodiesel. Appl. Sci..

[15] Schlesinger H.I., Brown H.C., Finholt E., Gilbreath J.R., Hoekstra H.R., Hyde E.K. (1953). Sodium Borohydride, Its Hydrolysis and its Use as a Reducing Agent and in the Generation of Hydrogen. J. Am. Chem. Soc..

[16] Quach Q., Biehler E., Elzamzami A., Huff C., Long J.M., Abdel-Fattah T.M. (2021). Catalytic Activity of Beta-Cyclodextrin-Gold Nanoparticles Network in Hydrogen Evolution Reaction. Catalysts.

[17] Huff C., Long J.M., Aboulatta A., Heyman A., Abdel-Fattah T.M. (2017). Silver Nanoparticle/Multi-Walled Carbon Nanotube Composite as Catalyst for Hydrogen Production. ECS J. Solid State Sci. Technol..

[18] Huff C., Biehler E., Quach Q., Long J.M., Abdel-Fattah T.M. (2021). Synthesis of Highly Dispersive Platinum Nanoparticles and their Application in a Hydrogen Generation Reaction. Colloids Surf. A Physicochem. Eng. Asp..

[19] Huff C., Long J.M., Heyman A., Abdel-Fattah T.M. (2018). Palladium Nanoparticle Multiwalled Carbon Nanotube Composite as Catalyst for Hydrogen Production by the Hydrolysis of Sodium Borohydride. ACS Appl. Energy Mater..

[20] Wittstock A., Neumann B., Schaefer A., Dumbuya K., Kübel C., Biener M.M., Zielasek V., Steinrück H.P., Gottfried J.P., Biener J. (2009). Nanoporous Au: An Unsupported Pure Gold Catalyst?. J. Phys. Chem. C.

[21] Li Z., Fu J.-Y., Feng Y., Dong C.-K., Liu H., Du X.-W. (2019). A silver catalyst activated by stacking faults for the hydrogen evolution reaction. Nat. Catal..

[22] Periana R.A., Taube D.J., Gamble S., Taube H., Satoh T., Fujii H. (1998). Platinum Catalysts for the High-Yield Oxidation of Methane to a Methanol Derivative. Science.

[23] Xia R., Zhang S., Ma X., Jiao F. (2020). Surface-Functionalized Palladium Catalysts for Electrochemical CO_2_ Reduction. J. Mater. Chem. A.

[24] Dushatinski T., Huff C., Abdel-Fattah T.M. (2016). Characterization of electrochemically deposited films from aqueous and ionic liquid cobalt precursors toward hydrogen evolution reactions. Appl. Surf. Sci..

[25] Osborne J., Horton M.R., Abdel-Fattah T.M. (2020). Gold Nanoparticles Supported Over Low-Cost Supports for Hydrogen Generation from a Hydrogen Feedstock Material. ECS J. Solid State Sci. Technol..

[26] Huff C., Dushatinski T., Barzanjii A., Abdel-Fattah N., Barzanjii K., Abdel-Fattah T.M. (2017). Pretreatment of gold nanoparticle multi-walled carbon nanotube composites for catalytic activity toward hydrogen generation reaction. ECS J. Solid State Sci. Technol..

[27] Nandanapalli K.R., Mudusu D., Lee S. (2020). Defects-free single-crystalline zinc oxide nanostructures for efficient photoelectrochemical solar hydrogen generation. Int. J. Hydrogen Energy.

[28] Kumar S., Kumar A., Kumar A., Krishnan V. (2019). Nanoscale zinc oxide based heterojunctions as visible light active photocatalysts for hydrogen energy and environmental remediation. Catal. Rev.-Sci. Eng..

[29] Gao Z., Chen K., Wang L., Bai B., Liu H., Wang Q. (2020). Aminated flower-like ZnIn_2_S_4_ coupled with benzoic acid modified g-C_3_N_4_ nanosheets via covalent bonds for ameliorated photocatalytic hydrogen generation. Appl. Catal. B.

[30] Quach Q., Abdel-Fattah T.M. (2022). Silver Nanoparticles functionalized Nanoporous Silica Nanoparticle grown over Graphene Oxide for enhancing Antibacterial effect. Nanomaterials.

[31] Biehler E., Whiteman R., Lin P., Zhang K., Baumgart H., Abdel-Fattah T.M. (2020). Controlled Synthesis of ZnO Nanorods Using Different Seed Layers. ECS J. Solid State Sci. Technol..

[32] Dushatinski T., Abdel-Fattah T.M. (2015). Carbon Nanotube Composite Mesh Film with Tunable Optoelectronic Performance. ECS J. Solid State Sci. Technol..

[33] Abdel-Fattah T.M., Wixtrom A., Zhang K., Baumgart H. (2014). Highly Uniform Self-Assembled Gold Nanoparticles over High Surface Area Dense ZnO Nanorod Arrays as Novel Surface Catalysts *ECS J*. Solid State Sci. Technol..

[34] Abdel-Fattah T.M., Wixtrom A. (2014). Catalytic Reduction of 4-Nitrophenol Using Gold Nanoparticles Supported On Carbon Nanotubes. ECS J. Solid State Sci. Technol..

[35] Sabourault N., Mignani G., Wagner A., Mioskowski C. (2002). Platinum Oxide (PtO_2_): A Potent Hydrosilylation Catalyst. Org. Lett..

[36] Ely J.C., Neal C.R., Kulpa C.F., Schneegurt M.A., Seidler J.A., Jain J.C. (2001). Implications of Platinum-Group Element Accumulation along U.S. Roads from Catalytic-Converter Attrition. Envir. Sci. Technol..

[37] Abu-Reziq R., Wang D., Post M., Alper H. (2007). Platinum Nanoparticles Supported on Ionic Liquid-Modified Magnetic Nanoparticles: Selective Hydrogenation Catalysts. Adv. Synth. Catal..

[38] Fang B., Chaudhari N.K., Kim M.-S., Kim J.H., Yu J.-S. (2009). Homogeneous Deposition of Platinum Nanoparticles on Carbon Black for Proton Exchange Membrane Fuel Cell. J. Am. Chem. Soc..

[39] Huff C., Quach Q., Long J.M., Abdel-Fattah T.M. (2020). Nanocomposite Catalyst Derived from Ultrafine Platinum Nanoparticles and Carbon Nanotubes for Hydrogen Generation. ECS J. Solid State Sci. Technol..

[40] Liu J., Zhou Z., Zhao X., Xin Q., Sun G., Yi B. (2004). Studies on Performance Degradation of a Direct Methanol Fuel Cell (DMFC) in Life Test. Phys. Chem. Chem. Phys..

[41] Zhu Y., Murali S., Cai W., Li X., Suk J.W., Potts J.R., Ruoff R.S. (2010). Graphene and Graphene Oxide: Synthesis, Properties, and Applications. Adv. Mater..

[42] Li K., Liu Q., Cheng H., Hu M., Zhang S. (2020). Classification and carbon structural transformation from anthracite to natural coaly graphite by XRD, Raman spectroscopy, and HRTEM. Spectrochim. Acta A.

[43] Kaufman C.M., Sen B. (1985). Hydrogen generation by hydrolysis of sodium tetrahydroborate: Effects of acids and transition metals and their salts. J. Chem. Soc. Dalton Trans..

[44] Narasimharao K., Abu-Zied B.M., Alfaifi S.Y. (2020). Cobalt oxide supported multi wall carbon nanotube catalysts for hydrogen production via sodium borohydride hydrolysis. Int. J. Hydrogen Energy.

[45] Saka C., Salih Eygi M., Balbay A. (2020). CoB doped acid modified zeolite catalyst for enhanced hydrogen release from sodium borohydride hydrolysis. Int. J. Hydrogen Energy.

[46] Lin K.-Y.A., Chang H.-A. (2016). Efficient hydrogen production from NaBH_4_ hydrolysis catalyzed by a magnetic cobalt/carbon composite derived from a zeolitic imidazolate framework. Chem. Eng. J..

[47] Peña-Alonso R., Sicurelli A., Callone E., Carturan G., Raj R. (2007). A picoscale catalyst for hydrogen generation from NaBH_4_ for fuel cells. J. Power Sources.

[48] Huff C., Dushatinski T., Abdel-Fattah T.M. (2017). Gold Nanoparticle/Multi-Walled Carbon Nanotube Composite as Novel Catalyst for Hydrogen Evolution Reactions. Int. J. Hydrogen Energy.

[49] Biehler E., Quach Q., Huff C., Abdel-Fattah T.M. (2022). Organo-Nanocups Assist the Formation of Ultra-Small Palladium Nanoparticle Catalysts for Hydrogen Evolution Reaction. Materials.

[50] Huff C., Long J.M., Abdel-Fattah T.M. (2020). Beta-Cyclodextrin Assisted Synthesis of Silver Nanoparticle Network and its Application in a Hydrogen Generation Reaction. Catalysts.

[51] Biehler E., Quach Q., Abdel-Fattah T.M. (2023). Silver Nanoparticle-Decorated Fused Carbon Sphere Composite as a Catalyst for Hydrogen Generation. Energies.

[52] Guella G., Patton B., Miotello A. (2007). Kinetic Features of the Platinum Catalyzed Hydrolysis of Sodium Borohydride from 11B NMR Measurements. J. Phys. Chem. C.

[53] Zhang H., Feng X., Cheng L., Hou X., Li Y., Han S. (2018). Non-noble Co anchored on nanoporous graphene oxide, as an efficient and long-life catalyst for hydrogen generation from sodium borohydride. Colloids Surf..

[54] Deraedt C., Salmon L., Gatard S., Ciganda R., Hernandez E., Ruiz J., Astruc D. (2014). Sodium borohydride stabilizes very active gold nanoparticle catalysts. Chem. Commun..

[55] Fang Y., Zhang J., Hua M.Y., Zhou D.W. (2020). Modifying effects and mechanisms of graphene on dehydrogenation properties of sodium borohydride. J. Mater. Sci..

