# Supplementary material for: Synthesis of Platinum Nanoparticles Supported on Fused Nanosized Carbon Spheres Derived from Sustainable Source for Application in a Hydrogen Generation Reaction
Source: Nanomaterials (Basel). 2023 Jul 1;13(13):1994. doi: 10.3390/nano13131994 (PMC10343661; doi:10.3390/nano13131994)
Supplement: Supplementary file 1 [file nanomaterials-13-01994-s001.zip › nanomaterials-2448196-supplementary.pdf]

Supplementary Material

Synthesis of Platinum Nanoparticles Supported on Fused nanosized Carbon Spheres derived from Sustainable source for Application in a Hydrogen Generation Reaction

Erik Biehler [1,2], Qui Quach [1,2] and Tarek M. Abdel-Fattah [1,2,*]

[1] Applied Research Center, Thomas Jefferson National Accelerator Facility, Newport News, VA 23606, USA; erik.biehler.16@cnu.edu (E.B.); qui.quach.13@cnu.edu (Q.Q.)
2
[2] Department of Molecular Biology and Chemistry, Christopher Newport University, Newport News, VA 23606, USA

[*]Correspondence: fattah@cnu.edu

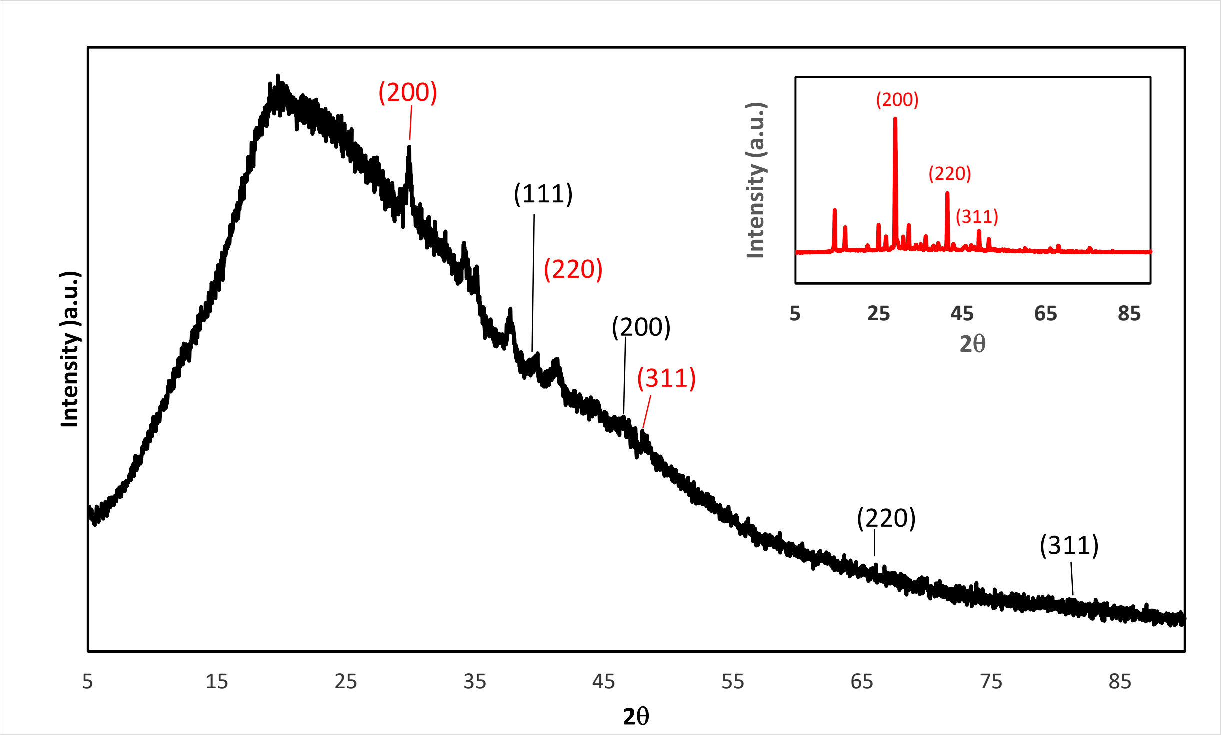

Figure S1: P-XRD of PtFCS after reusability trials. The inset showed the P-XRD of sodium borohydride. The red color showed the signature peaks of sodium borohydride.

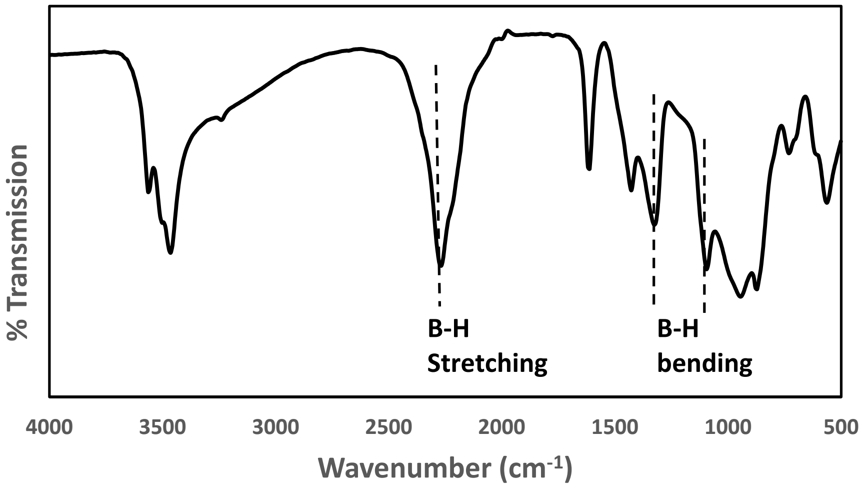

Figure S2: FTIR of sodium borohydride ($NaBH_4$)

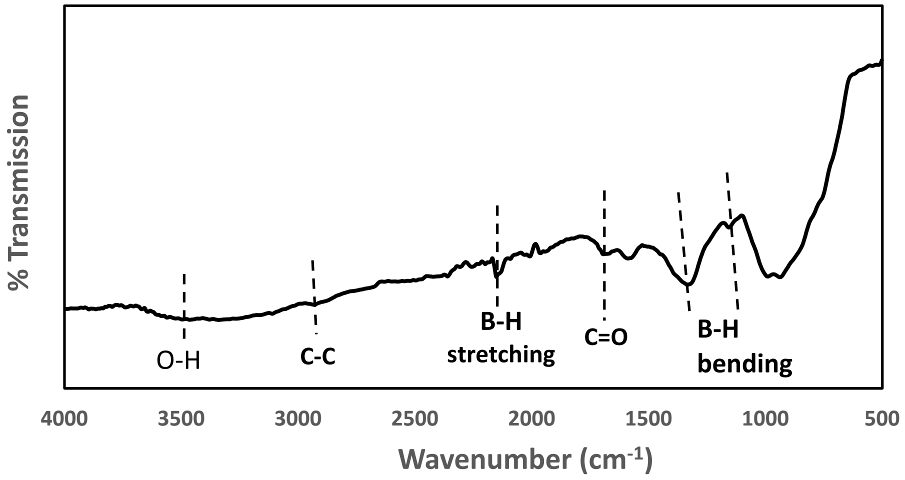

Figure S3: FTIR of PtFCS after reusability trials

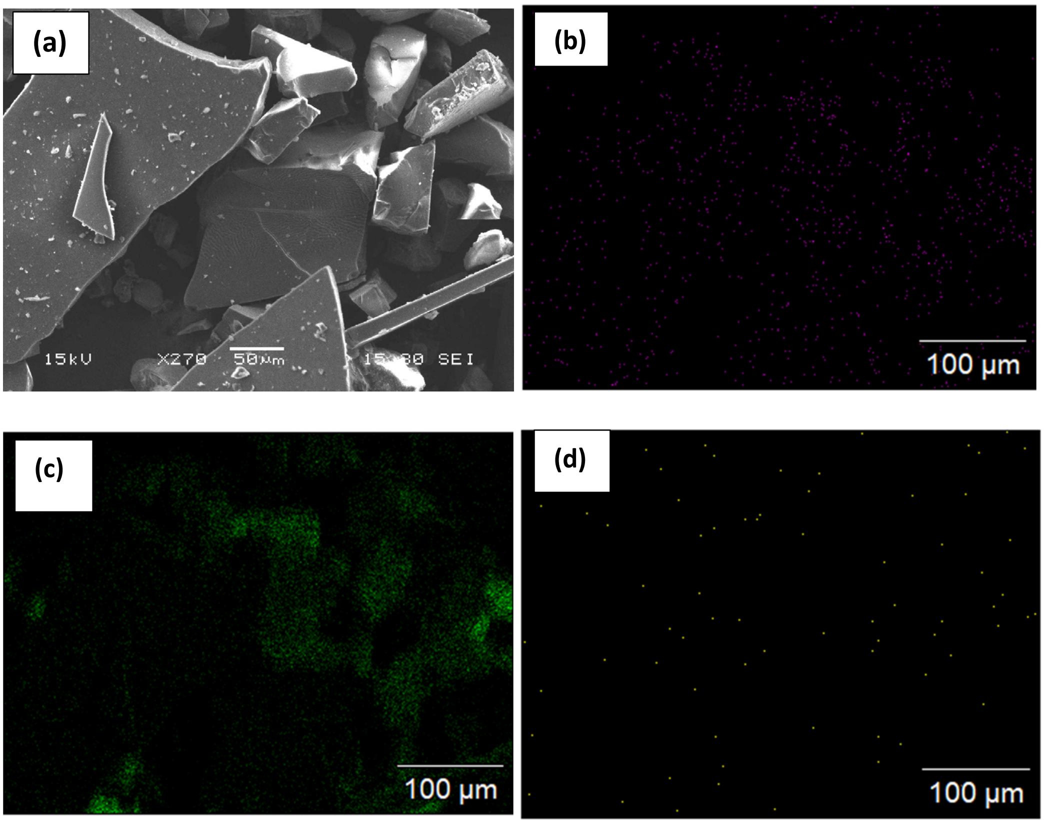

Figure S4: (a) SEM of PtFCS after reusability trials; (b) mapping of boron (B); (c) mapping of carbon (C); (d) mapping of platinum (Pt)
